# Supplementary figures and images for: Gametocyte production and transmission fitness of African and Asian Plasmodium falciparum isolates with differential susceptibility to artemisinins
Source: Antimicrob Agents Chemother. 2025 Apr 22;69(6):e01930-24. doi: 10.1128/aac.01930-24 (PMC12135531; doi:10.1128/aac.01930-24)

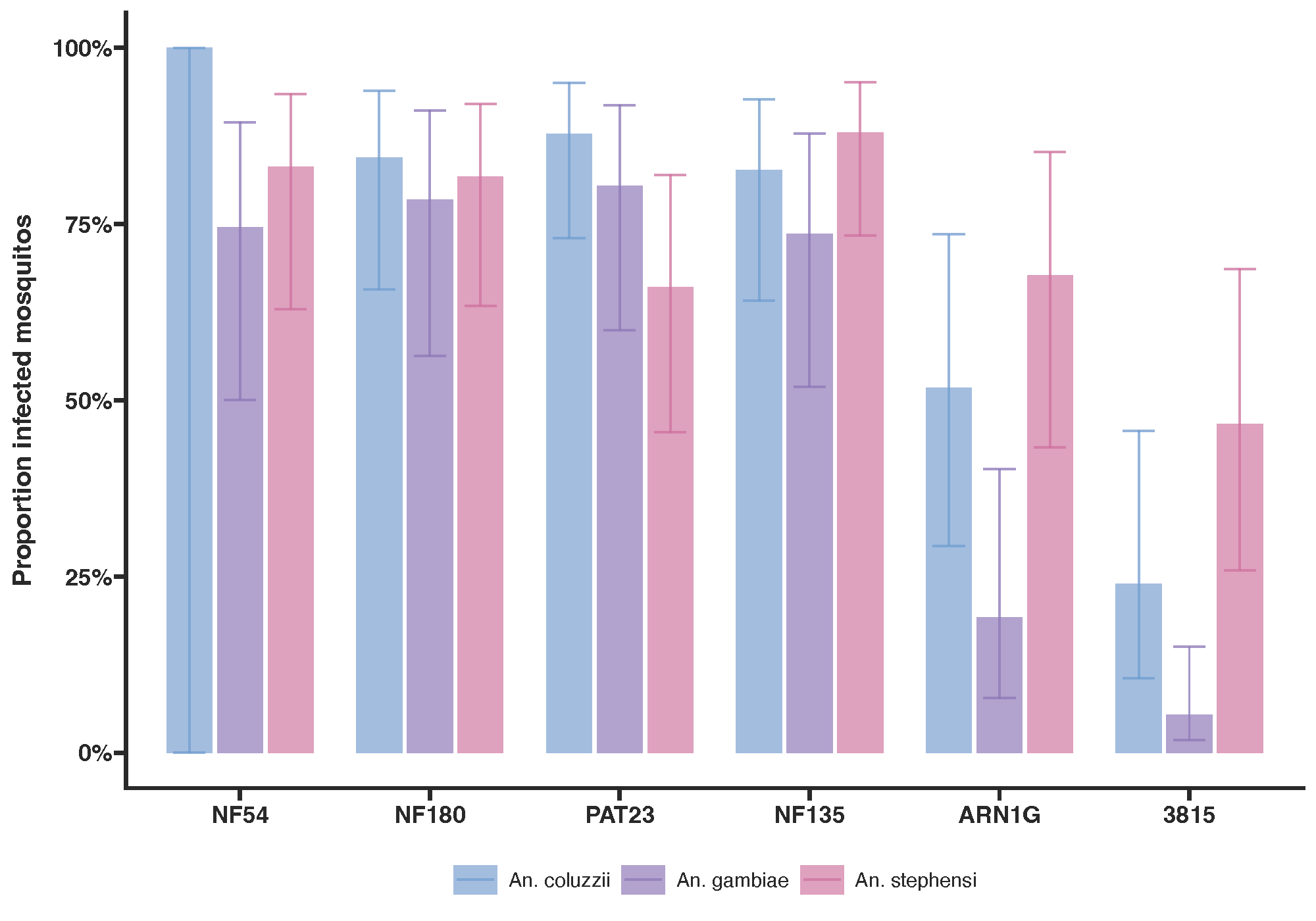

Supplement: Fig. S1 — Mosquito infection rates. [file aac.01930-24-s0001.tiff]

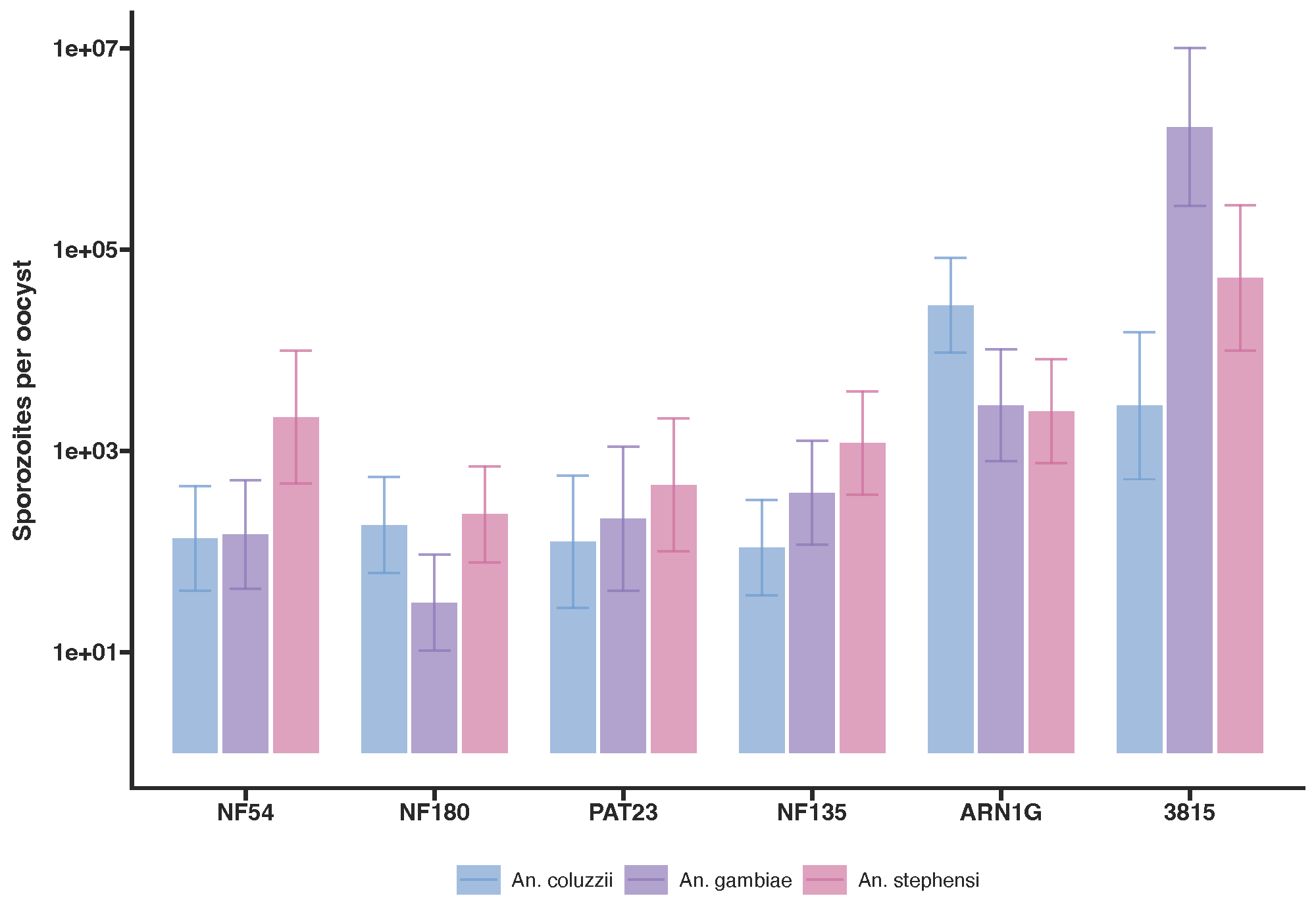

Supplement: Fig. S2 — Calculated sporozoite per oocyst. [file aac.01930-24-s0002.tiff]

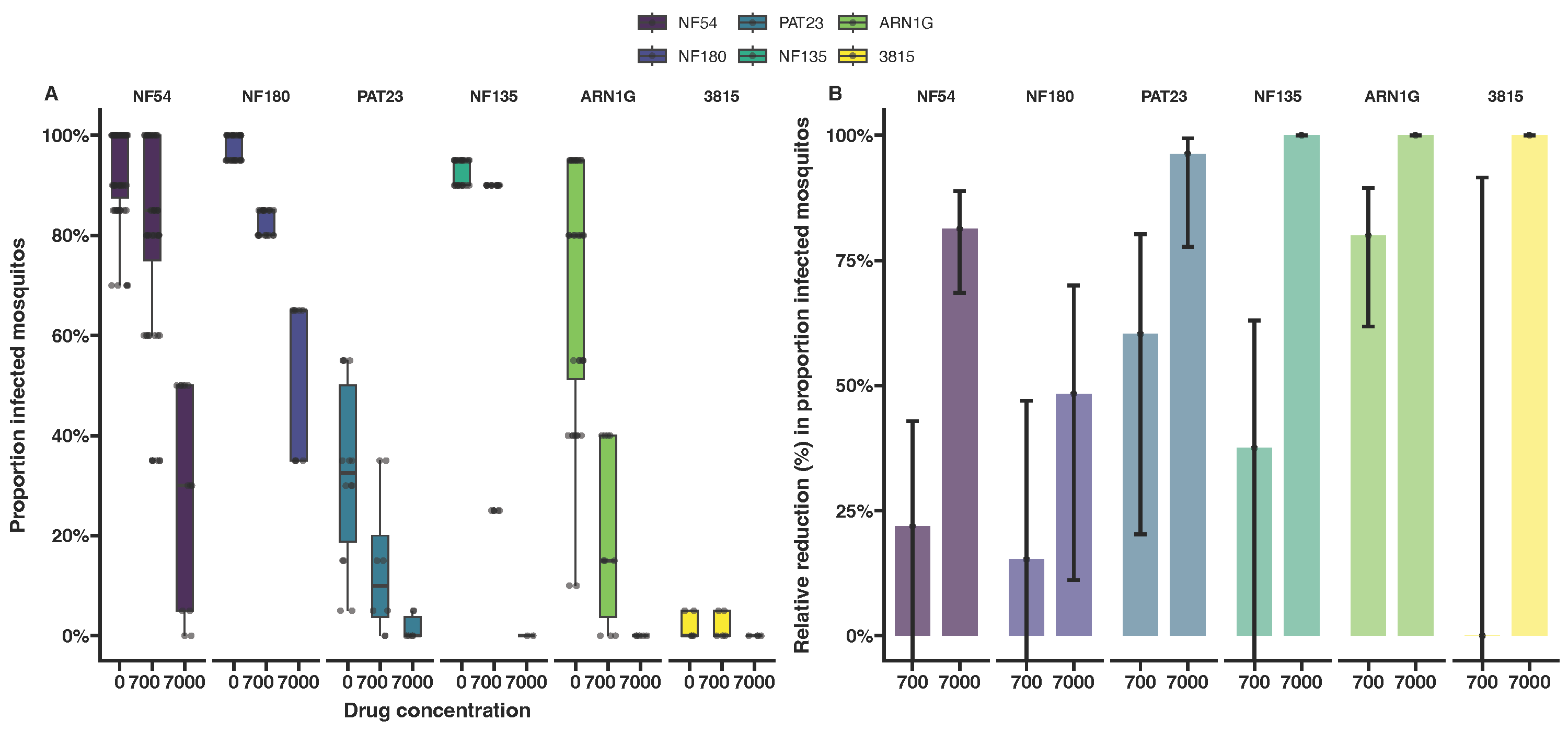

Supplement: Fig. S3 — Reduction in the overall oocyst prevalence following exposure to DHA. [file aac.01930-24-s0003.tiff]
