# Supplementary material for: Gametocyte production and transmission fitness of African and Asian Plasmodium falciparum isolates with differential susceptibility to artemisinins
Source: Antimicrob Agents Chemother. 2025 Apr 22;69(6):e01930-24. doi: 10.1128/aac.01930-24 (PMC12135531; doi:10.1128/aac.01930-24)
Supplement: Supplemental material — Tables S1 to S5; Supplemental figure legends. [file aac.01930-24-s0004.docx]

**Supplemental Tables**

**Sup Table 1. RSA comparisons**

| Reference parasite line | Parasite line | Ratio (CI) |
| --- | --- | --- |
| NF54 | NF180 | 0.99 (0.25, 3.85); p=0.9742 |
| NF54 | PAT-023 | 33.34 (10, 100); p<0.0001 |
| NF54 | NF135 | 6.67 (1.49, 33.34); p=0.0023 |
| NF54 | ARN1G | 20.00 (6.25, 100); p<0.0001 |
| NF54 | 3815 | 33.34 (12.5, 100); p<0.0001 |
|  |  |  |
| NF135 | PAT-023 | 4.31 (1.20, 15.49); p=0.0057 |
| NF135 | ARN1G | 3.13 (0.81, 12.5); p=0.0636 |
| NF135 | 3815 | 5.26 (1.51, 20); p=0.0014 |

**Sup Table 2. Sexual conversion comparisons**

| Parasite line | Reference media | Media | Ratio (CI) |
| --- | --- | --- | --- |
| NF54 | serum | mFa | 114.14 (31.57, 412.62); p<0.0001 |
| NF54 | serum | Albumax | 57.67 (15.77, 210.87); p<0.0001 |
| NF54 | mFa | Albumax | 0.51 ( 0.16, 1.60); p=0.1516 |
| NF180 | serum | mFa | 46.96 (14.23, 154.93); p<0.0001 |
| NF180 | serum | Albumax | 21.99 ( 6.47, 74.73); p<0.0001 |
| NF180 | mFa | Albumax | 0.47 ( 0.16, 1.39); p=0.0934 |
| PAT-023 | serum | mFa | 3.95 ( 1.06, 14.70); p=0.0374 |
| PAT-023 | serum | Albumax | 2.90 ( 0.78, 10.79); p=0.1027 |
| PAT-023 | mFa | Albumax | 0.73 ( 0.20, 2.74); p=0.5691 |
| NF135 | serum | mFa | 92.75 (25.51, 337.27); p<0.0001 |
| NF135 | serum | Albumax | 27.16 ( 6.95, 106.13); p<0.0001 |
| NF135 | mFa | Albumax | 0.29 ( 0.09, 1.01); p=0.0172 |
| ARN1G | serum | mFa | 75.74 (22.86, 250.88); p<0.0001 |
| ARN1G | serum | Albumax | 30.40 ( 8.75, 105.61); p<0.0001 |
| ARN1G | mFa | Albumax | 0.40 ( 0.13, 1.24); p=0.0511 |
| 3815 | serum | mFa | 7.27 ( 1.97, 26.84); p=0.0011 |
| 3815 | serum | Albumax | 4.30 ( 1.16, 16.00); p=0.0162 |
| 3815 | mFa | Albumax | 0.59 ( 0.16, 2.19); p=0.3311 |

mFa, minimal fatty acid.

**Sup Table 3. Oocyst comparisons**

| Parasite line | Reference mosquito species | Mosquito species | Oocyst ratio |
| --- | --- | --- | --- |
| NF54 | *An. stephensi* | *An. coluzzii* | 4.35 (2.5, 7.69); p<0.0001 |
| NF54 | *An. stephensi* | *An. gambiae* | 1.56 (0.87, 2.78); p=0.0695 |
| NF54 | *An. coluzzii* | *An. gambiae* | 0.35 (0.20, 0.62); p<0.0001 |
| NF180 | *An. stephensi* | *An. coluzzii* | 1.66 (1.01, 2.70); p=0.0285 |
| NF180 | *An. stephensi* | *An. gambiae* | 2.17 (1.33, 3.57); p=0.0005 |
| NF180 | *An. coluzzii* | *An. gambiae* | 1.31 (0.81, 2.12); p=0.1829 |
| PAT-023 | *An. stephensi* | *An. coluzzii* | 6.25 (3.45, 11.11); p<0.0001 |
| PAT-023 | *An. stephensi* | *An. gambiae* | 3.45 (1.96, 5.88); p<0.0001 |
| PAT-023 | *An. coluzzii* | *An. gambiae* | 0.55 (0.32, 0.94); p=0.0079 |
| NF135 | *An. stephensi* | *An. coluzzii* | 1.09 (0.66, 1.79); p=0.6706 |
| NF135 | *An. stephensi* | *An. gambiae* | 0.69 (0.42, 1.12); p=0.1353 |
| NF135 | *An. coluzzii* | *An. gambiae* | 0.63 (0.38, 1.03); p=0.0732 |
| ARN1G | *An. stephensi* | *An. coluzzii* | 0.53 (0.31, 0.9); p=0.0087 |
| ARN1G | *An. stephensi* | *An. gambiae* | 0.30 (0.18, 0.53); p<0.0001 |
| ARN1G | *An. coluzzii* | *An. gambiae* | 0.57 (0.32, 1.00); p=0.0167 |
| 3815 | *An. stephensi* | *An. coluzzii* | 0.44 (0.22, 0.9); p=0.0066 |
| 3815 | *An. stephensi* | *An. gambiae* | 0.08 (0.03, 0.24); p<0.0001 |
| 3815 | *An. coluzzii* | *An. gambiae* | 0.18 (0.06, 0.56); p=0.0006 |

**Sup Table 4. Sporozoite comparisons**

| Parasite line | Reference mosquito species | Mosquito species | Ratio (CI) |
| --- | --- | --- | --- |
| NF54 | *An. stephensi* | *An. coluzzii* | 0.28 (0.04, 2.0); p=0.2362 |
| NF54 | *An. stephensi* | *An. gambiae* | 0.11 (0.01, 0.79); p=0.0236 |
| NF54 | *An. coluzzii* | *An. gambiae* | 0.38 (0.07, 2.02); p=0.2362 |
| NF180 | *An. stephensi* | *An. coluzzii* | 1.30 (0.31, 5.26); p=0.6562 |
| NF180 | *An. stephensi* | *An. gambiae* | 0.28 (0.07, 1.19); p=0.0702 |
| NF180 | *An. coluzzii* | *An. gambiae* | 0.22 (0.05, 0.92); p=0.0333 |
| PAT-023 | *An. stephensi* | *An. coluzzii* | 1.69 (0.17, 16.67); p=1.0000 |
| PAT-023 | *An. stephensi* | *An. gambiae* | 1.59 (0.14, 16.67); p=1.0000 |
| PAT-023 | *An. coluzzii* | *An. gambiae* | 0.93 (0.08, 10.51); p=1.0000 |
| NF135 | *An. stephensi* | *An. coluzzii* | 0.09 (0.02, 0.46); p=0.0010 |
| NF135 | *An. stephensi* | *An. gambiae* | 0.22 (0.04, 1.09); p=0.0488 |
| NF135 | *An. coluzzii* | *An. gambiae* | 2.20 (0.48, 10.04); p=0.2114 |
| ARN1G | *An. stephensi* | *An. coluzzii* | 5.88 (1.35, 25.0); p=0.0084 |
| ARN1G | *An. stephensi* | *An. gambiae* | 0.35 (0.06, 1.92); p=0.1388 |
| ARN1G | *An. coluzzii* | *An. gambiae* | 0.06 (0.01, 0.29); p<0.0001 |
| 3815 | *An. stephensi* | *An. coluzzii* | 0.02 (0, 0.31); p=0.0011 |
| 3815 | *An. stephensi* | *An. gambiae* | 2.56 (0.2, 33.34); p=0.3758 |
| 3815 | *An. coluzzii* | *An. gambiae* | 106.97 (8.32, 1375.20); p<0.0001 |

**Sup Table 5. Sporozoite per oocyst comparisons**

| Parasite line | Reference mosquito species | Mosquito species | Sporozoite per oocyst ratio |
| --- | --- | --- | --- |
| NF54 | *An. stephensi* | *An. coluzzii* | 0.06 (0.01, 0.50); p=0.0044 |
| NF54 | *An. stephensi* | *An. gambiae* | 0.07 (0.01, 0.56); p=0.0047 |
| NF54 | *An. gambiae* | *An. coluzzii* | 0.92 (0.16, 5.33); p=0.9092 |
| NF180 | *An. stephensi* | *An. coluzzii* | 0.78 (0.17, 3.51); p=0.6919 |
| NF180 | *An. stephensi* | *An. gambiae* | 0.13 (0.03, 0.60); p=0.0042 |
| NF180 | *An. gambiae* | *An. coluzzii* | 5.89 (1.32, 26.36); p=0.0096 |
| PAT-023 | *An. stephensi* | *An. coluzzii* | 0.27 (0.03, 2.88); p=0.5525 |
| PAT-023 | *An. stephensi* | *An. gambiae* | 0.46 (0.04, 5.59); p=0.9104 |
| PAT-023 | *An. gambiae* | *An. coluzzii* | 0.59 (0.05, 7.06); p=0.9104 |
| NF135 | *An. stephensi* | *An. coluzzii* | 0.09 (0.02, 0.45); p=0.0012 |
| NF135 | *An. stephensi* | *An. gambiae* | 0.32 (0.06, 1.74); p=0.1198 |
| NF135 | *An. gambiae* | *An. coluzzii* | 0.29 (0.06, 1.42); p=0.1198 |
| ARN1G | *An. stephensi* | *An. coluzzii* | 11.30 (2.31, 55.28); p=0.0009 |
| ARN1G | *An. stephensi* | *An. gambiae* | 1.15 (0.19, 6.97); p=0.8499 |
| ARN1G | *An. gambiae* | *An. coluzzii* | 9.81 (1.79, 53.69); p=0.0028 |
| 3815 | *An. stephensi* | *An. coluzzii* | 0.05 (0.00, 0.76); p=0.0084 |
| 3815 | *An. stephensi* | *An. gambiae* | 31.40 (1.95, 504.25); p=0.0062 |
| 3815 | *An. gambiae* | *An. coluzzii* | 0.00 (0.00, 0.03); p<0.0001 |

**Supplemental Figure Legends**

**Sup Figure 1.** Mosquito infection rates for the six parasite lines in each mosquito species across three to four independent mosquito feeding experiments.

**Sup Figure 2.** Calculated sporozoite per oocyst**.** Sporozoites per salivary gland from individual mosquitoes were dissected and processed for qPCR. The sporozoites per oocysts was calculated based on the cage matched oocysts per mosquito average. The error bars represent the confidence intervals.

**Sup Figure 3.** Reduction in the overall oocyst prevalence of the six parasites lines following exposure to DHA. **A)** Shows the decrease in mosquito infectivity upon exposing mature gametocytes to 700nM and 7000nM DHA prior to being fed to mosquitoes in a blood meal. The dots represent the prevalence from an individual mosquito cage. **B)** Represents the relative reduction in oocysts density compared the no drug control. All error bars represent the confidence intervals.
